# A new protocol for psychrometric pressure–volume curves of fern gametophytes

**DOI:** 10.1002/aps3.1248

**Published:** 2019-05-10

**Authors:** Christopher P. Krieg, James E. Watkins, Katherine A. McCulloh

**Affiliations:** ^1^ Department of Biology University of Florida Gainesville Florida 32611 USA; ^2^ Department of Biology Colgate University Hamilton New York 13346 USA; ^3^ Department of Botany University of Wisconsin Madison Wisconsin 35706 USA

**Keywords:** ferns, gametophytes, plant–water relations, psychrometry, turgor loss point

## Abstract

**Premise:**

Pressure**–**volume curves are a widely used analytical framework to derive several key physiological traits related to plant–water relations, including a species’ turgor loss point, osmotic potential at full turgor, and the elasticity of cell walls. We developed a novel protocol, including the preparation and treatment of fern gametophytes, to generate data for pressure**–**volume curve analyses using thermocouple psychrometry.

**Methods and Results:**

Gametophytes of the fern species *Polystichum lemmonii* were grown from spore, harvested, and subjected to a series of drying intervals. We constructed pressure–volume curves using thermocouple psychrometers to calculate gametophyte water potential and a balance to measure relative water loss.

**Conclusions:**

We present the first protocol for fern gametophyte pressure–volume curves that can accurately determine key physiological traits in fern gametophytes such as the turgor loss point and osmotic potential at full turgor.

Plant responses to water stress are among the most widely measured physiological traits in the field of plant physiological ecology. One of the most useful and common approaches in plant–water relations research is the pressure–volume curve (PV curve), which describes the relationship between decreasing water potential (Ψ) and relative water content (RWC). Several predictive parameters can be derived from a plant PV curve such the turgor loss point (Ψ_tlp_; i.e., the water potential at which the pressure potential = 0 MPa), osmotic potential at full turgor (π_o_), and bulk elastic modulus (ε) of cells (Tyree and Hammel, 1972). Numerous studies have shown strong correlations between aspects of a species’ ecological niche and the species’ Ψ_tlp_, π_o_, and ε (Lenz et al., 2006; Baltzer et al., 2008; Li et al., 2018). Moreover, published Ψ_tlp_ data are available for nearly every major lineage of vascular plants, and recent meta‐analyses of published data show this trait is at the nexus of many physiological and ecological processes (Bartlett et al., 2012; Zhu et al., 2018).

Ferns are the second most diverse group of vascular plants (PPG I, 2016) and have long been of interest to botanists because of their unique physiology and ecological diversity (Campbell, 1948; Smith, 1972; Nobel, 1978; Calkin et al., 1985; Brodribb and McAdam, 2011; Watkins and Cardelús, 2012; Pittermann et al., 2015). Similar to studies of other plant groups, PV curve studies of fern sporophytes have shown that traits such as Ψ_tlp_ can be strong predictors of drought tolerance and ecological niche (Proctor, 2003; Lo Gullo et al., 2010). However, recruitment in ferns begins with the gametophyte generation. This stage of the fern life cycle is markedly different from the sporophyte stage, creating a radical system of biology where two phases of an organism's life cycle are under different and potentially opposite selective pressures. Specifically, and in contrast to sporophytes, fern gametophytes are a single cell layer thick and have a poorly developed cuticle, no vascular tissue, and no stomata. Therefore, the drought tolerance of fern gametophytes is largely controlled by the dynamics of cell water retention and release and influenced by a number of physical and chemical factors that may vary among species and locally adapted populations including the osmotic potential of cells, cell size, and the elasticity of cell walls (Tyree and Hammel, 1972; Steudle et al., 1977; Bartlett et al., 2012). Despite recent gains in our knowledge of fern gametophyte physiology that clearly demonstrate the ecological and evolutionary significance of gametophyte biology (Watkins, 2006; Watkins et al., 2007; Watkins and Cardelús, 2012), the dynamics of cell water retention and release in fern gametophytes remain largely unexplored. Psychrometric protocols have been extensively used to understand aspects of leaf–water relations and have been particularly useful to study seed‐free plants including multicellular, proto‐vascular gametophytes (e.g., hydroid‐bearing mosses), lichens, liverworts, and algae (Santarius, 1994; Beckett et al., 1997; Proctor et al., 1998). Such studies demonstrate the power and adaptability of thermocouple psychrometry to generate PV curves for diverse organismal systems. Hence, the lack of protocols for generating PV curves specifically for fern gametophytes significantly limits our ability to explore the aspects of plant cell–water relations (e.g., Ψ_tlp_, π_o_, and ε) for which PV curves have been so widely used in other organismal systems.

To date, all PV curve studies of ferns have been conducted on the sporophyte life stage. Traditionally, water potential measurements for PV curves are made using a Scholander–Hammel pressure chamber, which cannot be used for gametophytes. The Scholander–Hammel pressure chamber applies increasingly positive air pressure to plant tissue (e.g., a detached leaf with an exposed petiole for viewing) to find the positive pressure that is equal and opposite the negative pressure (i.e., water potential) of the sample. The lack of PV curve analyses available on fern gametophytes may further be due to the difficulty of working with these organisms (e.g., small size and fragility) or to a lack of recent interest in gametophyte biology.

The first studies to examine water stress responses in fern gametophytes were from F. L. Pickett, who used a variety of methods to induce gametophyte desiccation in open air and using desiccants (Pickett, 1913, 1914). The majority of recent studies examining fern gametophyte–water relations have utilized a series of salt desiccants, which involves placing gametophytes in sealed containers above saturated salt solutions that produce a known vapor pressure deficit (Rockland, 1960). Over a period of time, gametophytes come into equilibrium with the air (at atmospheric pressure) in the sealed chamber, thus allowing for the calculation of whole gametophyte water potential from the equilibrium vapor pressure. Although the salt method and psychrometric methods differ in how the equilibrium vapor pressure is estimated, both methods calculate whole gametophyte water potential using the thermodynamic equation:

$$\Psi =\left(RT/Vw\right)\;\text{ln}\left(p/p_{o}\right)$$

where Ψ is water potential (MPa), *R* is the universal gas constant (8.314 × 10^−6^ MJ mol^−1^·K^−1^), *T* is temperature (K), *Vw* is molar volume of water (1.8 × 10^−5^ m^3^·mol^−1^), and *p*/*p*_*o*_ is relative humidity expressed as a fraction where *p* is actual vapor pressure of air in equilibrium with the liquid phase (MPa) and *p*_*o*_ is saturation vapor pressure (MPa) at *T*. From the equation above, it is clear that both methods are highly sensitive to temperature. However, this is especially true for the salt method, which does not typically implement explicit steps for temperature stabilization and requires a longer equilibration time (thus being exposed to greater natural temperature fluctuations). Although desiccant methods, and the salt method in particular, have produced insights into gametophyte biology, they do not easily allow for the determination of typical PV curve parameters. Here, we present a full protocol (Appendix 1) for conducting PV curves with fern gametophytes using thermocouple psychrometer chambers.

## METHODS AND RESULTS

### Spore cultures

Spore samples are collected from three mature fern sporophytes of *Polystichum lemmonii* Underw. at Umatilla National Forest in Oregon, USA (44.783°N, 118.625°W), and Wenatchee National Forest in Washington, USA (47.411°N, 120.908°W). Spore material from each site are sown in Petri dishes (ThermoFisher Scientific, Waltham, Massachusetts, USA) containing Bold's medium (Bold, 1957) modified with Nitsch's micronutrients (Nitsch, 1951). Petri plates are kept at 23–26°C and exposed to a 10 h : 14 h, light : dark cycle achieved with fluorescent grow bulbs (ca. 50 μmol·m^2^·s) in a CMP 3244 growth chamber (Conviron Ltd., Winnipeg, Canada) at the University of Wisconsin–Madison. An excellent review of fern gametophyte propagation and spore culturing techniques is available in Raghavan (1989).

### Gametophyte preparation

Once spores grow into mature gametophytes, approximately 15–20 gametophytes are selected per plate, with a total gametophyte cluster mass between 40 and 67 mg. Given the relatively small size and weights of fern gametophytes, we recommend using a balance with a precision of at least ±0.1 mg (e.g., we used Entris 224 [Sartorius, Göttingen, Germany]). The clusters of gametophytes are transferred to a new Petri dish lined with moist filter paper. The moist filter paper is used to prevent unwanted desiccation (paper towel may also be used). The gametophytes should be thoroughly examined under a dissecting microscope to ensure there is no debris or agar. If there is debris or agar, try removing it with fine‐tip forceps (debris) or a wet fine‐tip paintbrush (agar). Be sure to have prepared several new Petri dishes with moist filter paper prior to the next step; each gametophyte cluster is subjected to a vigorous rinse with deionized water for ca. 30 s on a fresh Petri plate with moistened filter paper. Gametophyte clusters are then transferred to a second Petri dish with moistened filter paper and repeated (we recommend at least three rinses). The rinses further ensure that no debris or agar remains on the gametophytes. After the rinsing cycles, the gametophyte cluster (hereafter sample) is transferred to dry filter paper and excess water is blotted with Kimwipes (Kimberly‐Clark, Irving, Texas, USA). We recommend performing this step under a dissecting microscope to ensure that all excess water is removed.

### Psychrometric protocol

The sample is immediately placed on the balance and the mass recorded (i.e., turgid mass). As quickly as possible, the sample is placed into the psychrometer chamber (75‐3V thermocouple psychrometer and 81‐250 chamber; JRD Merrill Specialty Equipment, Logan, Utah, USA) connected to a data logger (Model CR‐6; Campbell Scientific Inc., Logan, Utah, USA) with a multiplexer (AM 16/32; Campbell Scientific Inc.). These steps are repeated for each sample (i.e., dry, weigh, place in chamber). The sealed chambers are placed into closeable plastic bags and in a water bath for thermal stability and allowed to equilibrate with the chamber air space for ca. 3 h. Measurements of the chamber thermocouple in microvolts are made by the data logger (Campbell Scientific Inc.) every 25 min. Voltage outputs are converted to water potential values by calibrating each chamber using salt solutions prior to making measurements on samples (Brown and Bartos, 1982). Equilibration times are determined from periodic downloads and examination of the data by plotting time versus microvolts and looking for a clear plateau (indicating a stable chamber vapor pressure). We achieved equilibration in approximately 3 h, however, larger chamber spaces and smaller samples both increase the time required for chambers to equilibrate. Once equilibrated, samples are removed and their mass immediately recorded using the balance (i.e., fresh mass). It is important to record the mass immediately after removing the sample from the chamber to minimize any potential changes in water potential after removal from the chambers. The samples are then placed in a weighing tin and allowed to desiccate in the air at room temperature for a specified time (hereafter “drying interval”).

In the first drying interval, the gametophyte samples are weighed and then immediately returned to the chambers, ensuring their exposure to room air for ca. 10 min. We optimized the number and duration of drying time intervals to a total of nine intervals that ranged from less than 10 min in the first interval to 50 min in the last intervals. By the last interval, all samples should have 4–10% of their initial turgid weight remaining (Table 1). After the series of psychrometry measurements, each sample is placed in labeled, open microcentrifuge tubes and arranged in a microcentrifuge tube rack, and then placed into a paper bag before being stored in a drying oven for 36 h at ca. 75°C. The paper bag ensures that oven air currents do not blow debris into or gametophyte tissue out of the microcentrifuge tube. After 36 h, the oven door is opened and the microcentrifuge tubes are quickly closed while still on the oven shelf. Once closed, the tubes are transported to the balance to determine their dry weights. Importantly, samples are not allowed to cool to room temperature outside of the sealed tubes, because that would result in the reabsorption of water from the air and overestimate the dry weight.

**Table 1:**
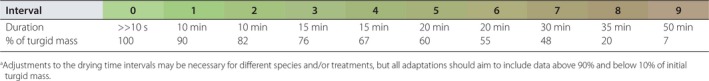
A generalized format for drying time intervals showing some expected relationships between interval number, interval duration, and percent of initial turgid mass.^a^

### Calculation of physiological parameters

Relative water content (RWC) of gametophytic tissue is calculated following the standard equation:

$$\begin{array}{cc} \text{RWC}\;(\%)= & \;[(\text{fresh weight}\;\;-\;\;\text{dry weight})\\ & \;/(\text{turgid weight}\;\;-\;\;\text{dry weight})]\times 100 \end{array}$$

**Figure 1:**
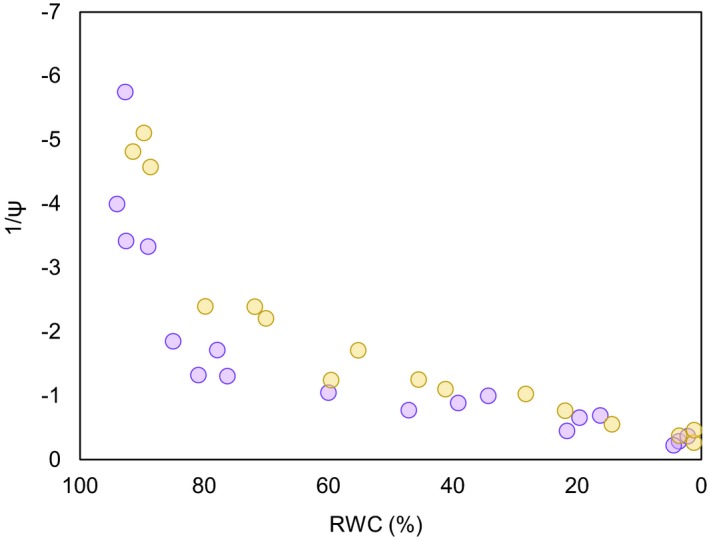
Pressure**–**volume curves (illustrated by individual points) showing the relationships between relative water content (RWC) and water potential (Ψ) in samples of *Polystichum lemmonii* taken from two sites in Umatilla National Forest (purple) and Wenatchee National Forest (yellow).

Physiological parameters (e.g., Ψ_tlp_, π_o_, ε) are derived from the relationship between water potential and relative water content data (e.g., Fig. 1) within the PV curve analytical framework following Tyree and Hammel (1972).

## CONCLUSIONS

Our detailed sample treatment and psychrometric protocol is the first of its kind for fern gametophytes and has several advantages over existing methods to explore fern gametophyte–water relations. For example, our psychrometric protocol, like other psychrometric protocols, allows for significantly greater control over temperature—the most difficult parameter to control with existing desiccant methods. In addition, gametophyte tissue generally takes significantly less time to equilibrate inside chamber psychrometers than containers with salt solutions that are typically larger (ca. 3 h and 24 h for chamber psychrometers and salt containers, respectively). Therefore, much more data can be gathered in a shorter time frame with our psychrometric protocol. Finally, in our view, the most significant advantage of our method is access to a new type of trait data for fern gametophytes (i.e., Ψ_tlp_, π_o_, ε). Hydraulic traits such as Ψ_tlp_, π_o_, and ε have been used in nearly every lineage of vascular plants to understand plant stress tolerance and aspects of species’ distributions and ecological niche (Lenz et al., 2006; Bartlett et al., 2012; McCulloh et al., 2014; Zhu et al., 2018). Until now, there have been no data available concerning these traits for fern gametophytes. Our detailed sample treatment and psychrometric protocol addresses this gap and offers key advances in gametophyte–water relations; however, interested researchers should first consider potential drawbacks and limitations. For example, researchers should remember that psychrometers and lab balances can be relatively expensive (the salt method also requires a balance). In addition, our protocol does not afford researchers the ability to predetermine the water potential of fern gametophyte tissue, although this is easily achievable with desiccant methods (e.g., the salt method). Researchers should also be aware that some work has shown that the rate of desiccation impacts recovery and that this impact is species specific (Watkins et al., 2007). If researchers are interested in recovery from desiccation, drying rate must be considered and would require modification of our protocol to manipulate drying rate during drying intervals, e.g., by covering gametophytes to slow water loss or using a series of salt desiccants to control water loss. Additionally, our psychrometric protocol may strongly complement the salt methods in some cases. For example, our psychrometric method can be used to understand plant–water relationships within the well‐established framework of PV curves, and summarized with the key physiological traits such as Ψ_tlp_, π_o_, and ε. Once a species’ Ψ_tlp_ is determined, a series of salt solutions could be optimized at precise water potentials at or bounding the Ψ_tlp_. Then, researchers could further investigate the significance of a gametophyte's Ψ_tlp_ with respect to traits such as photosynthetic activity, gene expression, sperm and egg production, fertilization, growth, and survival. We hope our protocol will be used to unlock these and many other avenues of research in fern gametophyte biology.

## AUTHOR CONTRIBUTIONS

C.P.K. and K.A.M. conceived of the idea. C.P.K. worked out the protocol with advice from K.A.M. and J.E.W. C.P.K. collected and analyzed the data. C.P.K., J.E.W., and K.A.M. wrote the manuscript.

## APPENDIX 1 Lab protocol for preparing gametophyte tissue and conducting pressure–volume curves for fern gametophytes.

### Parts list

- Balance with a precision of at least ±0.1 mg (Entris 224; Sartorius, Göttingen, Germany).
- Thermocouple psychrometer (75‐3V; JRD Merrill Specialty Equipment, Logan, Utah, USA) with small chamber (81‐250; JRD Merrill Specialty Equipment)
- Data logger (Model CR‐6; Campbell Scientific Inc., Logan, Utah, USA)
- Multiplexer (AM 16/32; Campbell Scientific Inc.).
- Forceps and fine‐tip brush
- Petri dishes
- Filter paper
- Dissecting scope
- Kimwipes (Kimberly‐Clark, Irving, Texas, USA)
- Weighing tin/boat
- Microcentrifuge tubes and rack
- Paper bag
- Drying oven
- Plastic bags
- A large insulated cooler

### Growing gametophytes

Note: Many protocols are available for growing gametophytes and can be used with this protocol (see Raghavan, 1989; Kumar et al., 2011).

### Sample preparation

1. We recommend working with spores from a maximum of one individual per Petri plate.
2. Once spores grow into mature gametophytes, select approximately 15–20 gametophytes per plate with a total gametophyte cluster mass between 40 and 67 mg.
3. Transfer each gametophyte cluster sample to a new Petri dish lined with moist filter paper.
4. Thoroughly examine under a dissecting microscope to ensure there is no debris or agar. If there is debris or agar, try removing it with fine‐tip forceps (debris) or a wet fine‐tip paintbrush (agar).
5. Rinse each gametophyte sample with deionized water for ca. 30 s on a fresh Petri plate with moistened filter paper.
6. Transfer each sample to a new Petri dish lined with moist filter paper and repeat (we recommend at least three rinses).
7. After the rinsing cycles, transfer the sample to dry filter paper.
8. Using a dissecting microscope, blot all excess water with Kimwipes.

### Psychrometric protocol

1. Immediately place the sample on the balance and record the mass (i.e., turgid fresh mass).
2. As quickly as possible, place the sample into the psychrometer chamber.
3. Place the sealed chambers into closeable plastic bags and in a water bath for thermal stability for ca. 3 h. (Check data logger data to make sure the sample has equilibrated.)
4. Once equilibrated, remove each sample and immediately record the mass of each sample (i.e., fresh mass).
5. Then place each sample in a weighing tin and allow it to desiccate in the air at room temperature until the sample has lost a maximum of 10% of its turgid mass (Table 1).
6. Repeat steps 1 through 4. Repeat step 5 for each sample and progressively allow samples to desiccate in accordance with Table 1. By the last interval, all samples should have lost 90% or more of their initial turgid weight (Table 1).
7. At the end of psychrometry measurements, place each sample in a labeled, open microcentrifuge tube and arrange tubes in a rack. Place the rack into a paper bag.
8. Store the covered samples in a drying oven for ca. 36 h at ca. 75°C.
9. After drying, open drying oven door and quickly close the microcentrifuge tubes.
10. Immediately record the dry weight of each sample on a balance (i.e., dry weight).

### Calculation of physiological parameters

1. Relative water content (RWC) is calculated following the standard equation:
  $$\begin{array}{cc} \text{RWC}\;(\%)=\; & \left[(\text{fresh weight}\;\;-\;\;\text{dry weight}\right)\\ & /(\text{turgid weight}\;\;-\;\;\text{dry weight})]\times 100 \end{array}$$
2. Physiological parameters (e.g., Ψ_tlp_, π_o_, ε) can be derived from the relationship between water potential and relative water content data (e.g., Fig. 1) within the pressure–volume curve analytical framework following Tyree and Hammel (1972).
